# High added value of a population-based participatory surveillance system for community acute gastrointestinal, respiratory and influenza-like illnesses in Sweden, 2013–2014 using the web

**DOI:** 10.1017/S0950268816003290

**Published:** 2017-01-31

**Authors:** A. PINI, H. MERK, A. CARNAHAN, I. GALANIS, E. VAN STRATEN, K. DANIS, M. EDELSTEIN, A. WALLENSTEN

**Affiliations:** 1 The Public Health Agency of Sweden, Stockholm, Sweden; 2 European Programme for Intervention Epidemiology Training (EPIET), European Centre for Disease Prevention and Control (ECDC), Stockholm, Sweden; 3 Santé Publique France, Public Health Agency, France; 4 Department of Medical Sciences, Uppsala University, Uppsala, Sweden

**Keywords:** Acute gastrointestinal illness, acute respiratory illness, influenza-like illness, population-based study, surveillance system

## Abstract

In 2013–2014, the Public Health Agency of Sweden developed a web-based participatory surveillance system, Hӓlsorapport, based on a random sample of individuals reporting symptoms weekly online, to estimate the community incidence of self-reported acute gastrointestinal (AGI), acute respiratory (ARI) and influenza-like (ILI) illnesses and their severity. We evaluated Hӓlsorapport's acceptability, completeness, representativeness and its data correlation with other surveillance data. We calculated response proportions and Spearman correlation coefficients (*r*) between (i) incidence of illnesses in Hӓlsorapport and (ii) proportions of specific search terms to medical-advice website and reasons for calling a medical advice hotline. Of 34 748 invitees, 3245 (9·3%) joined the cohort. Participants answered 81% (139 013) of the weekly questionnaires and 90% (16 351) of follow-up questionnaires. AGI incidence correlated with searches on winter-vomiting disease [*r* = 0·81, 95% confidence interval (CI) 0·69–0·89], and ARI incidence correlated with searches on cough (*r* = 0·77, 95% CI 0·62–0·86). ILI incidence correlated with the web query-based estimated incidence of ILI patients consulting physicians (*r* = 0·63, 95% CI 0·42–0·77). The high response to different questionnaires and the correlation with other syndromic surveillance systems suggest that Hӓlsorapport offers a reasonable representation of AGI, ARI and ILI patterns in the community and can complement traditional and syndromic surveillance systems to estimate their burden in the community.

## INTRODUCTION

Surveillance of common conditions in the general population, such as acute gastrointestinal illness (AGI), acute respiratory illness (ARI) and influenza-like illness (ILI), could provide estimates of the burden of these syndromes in the community, measure and facilitate assessment of the impact of public health policies, and help identify priorities triggering new interventions.

In Sweden, the Public Health Agency of Sweden (PHAS) manages and coordinates national infectious disease surveillance that is centred on the mandatory notifications of a broad spectrum of infectious diseases [1]. As a complement to this traditional surveillance, PHAS also employs syndromic surveillance systems based on non-specific symptoms or other health proxies that constitute a provisional diagnosis [2].

Two such syndromic surveillance systems use data from the national medical telephone advice hotline (1177) and specific queries to the national medical advice website (1177.se), respectively. The use of these systems is progressively increasing. As an example, in 2014, Get Well (webbsök), a web query-based algorithm of influenza-related symptoms that aims to estimate influenza activity, replaced the sentinel surveillance for ILI.

However, even if the traditional and syndromic surveillance systems allow surveillance of specific infectious agents and trends in activity, they cannot estimate the incidence of AGI, ARI and ILI in the community, since these systems are dependent on healthcare or health-information-seeking behaviour. Nonetheless, information on community incidence and severity may be crucial, not only when identifying general public health priorities, but also in case of health emergencies such as an influenza pandemic. In such circumstances, healthcare and health-information-seeking behaviour may change unpredictably and the interpretation of both the traditional and syndromic surveillance systems may be uncertain [3].

Recognizing these limitations, Sweden has tested and evaluated telephone- and web-based population-based surveillance [4, 5], but this effort was limited in the number of syndromes possible to include and its ability to add questionnaires. To enable feasible population-based surveillance of a broader spectrum of syndromes with the flexibility to add questionnaires, such as on disease severity, the PHAS set up Hӓlsorapport, a population-based surveillance system that entirely relies on symptoms reported over the web.

### Hӓlsorapport

In October 2013, the PHAS invited 34 748 people aged 3 months to 85 years of 34 842 eligible individuals selected from the Swedish population register, using age-stratified random sampling. Invitees received an invitation letter delivered through the national postal service to their registered residence. The number of invitees within each age group was calculated based on age-specific participation rates from previous PHAS web-based systems. For participants aged <16 years, the invitation was sent to the legal guardian who was asked to report on behalf of the child. Upon recruitment, participants completed an online intake questionnaire on sociodemographic characteristics. For participants aged <16 years, the guardians’ sociodemographic characteristics were collected.

From 18 November 2013 to 17 November 2014, participants were invited to answer weekly questionnaires investigating the new occurrence of 17 symptoms in the previous week (see Supplementary material). All participants who reported at least one symptom received a follow-up questionnaire 3½ weeks later investigating the severity of the episode and associated healthcare use. The follow-up questionnaires were sent until 11 December 2014.

Based on a previous study conducted in The Netherlands in 1998–1999 [6], AGI episodes were defined as episodes with at least three loose stools, or vomiting within 24 h, or loose stools/vomiting with at least two of the following additional symptoms: loose stool, vomiting, abdominal cramps, abdominal pain, fever, nausea, blood in the stool, or mucus in the stool, regardless if the case definitions for ARI or ILI were met in the same week. An ARI episode was defined as at least one of the following symptoms: cough, sore throat, shortness of breath, or coryza, if criteria for ILI were not met. Finally, based on the European definition, ILI episodes were defined as cough or sore throat or shortness of breath plus fever or headache or muscle ache or malaise plus a sudden onset of illness [7].

### Rationale of the evaluation and aims

In order to identify strengths and weaknesses of the project and advise stakeholders on its continuation, we assessed Hӓlsorapport's acceptability, completeness, and representativeness, its data correlation with data of other syndromic surveillance systems, and participants’ experience.

## METHODS

We defined participants as invitees who answered at least the intake questionnaire. For each weekly questionnaire, we defined responders as participants who answered the weekly questionnaire and active responders as responders who answered at least another weekly questionnaire in the previous 3 weeks.

### Data collection (Fig. 1)

All invitees received a unique identifier code to log-in to the cohort website and complete an online socio-demographic questionnaire. Once registered, participants’ answers to the emailed weekly online questionnaires were automatically saved on the server and were regularly exported as comma-separated value (.csv) files. Participants also received an email link to answer a follow-up questionnaire created using Survey Generator (http://www.alstra.se). These data were also automatically saved and regularly exported as .csv files.

**Fig. 1.**
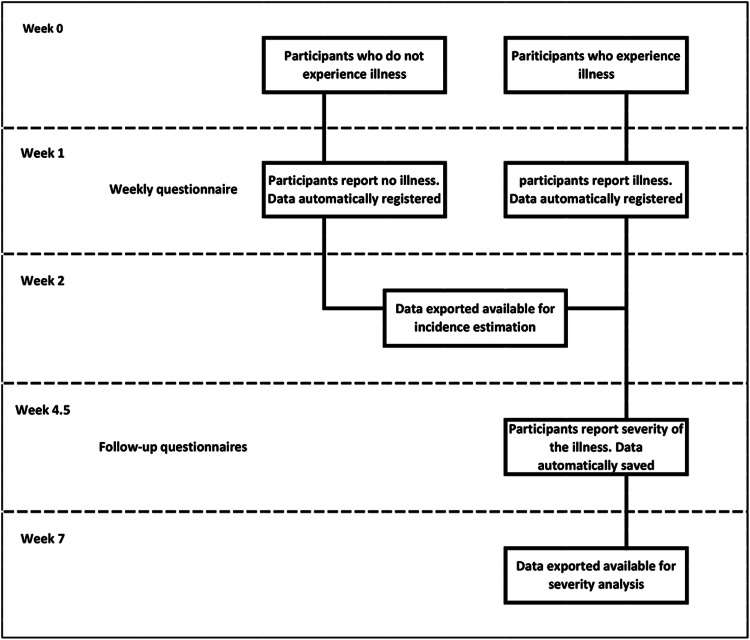
Data collection flow chart. Temporal representation of the data collection process in relation to the illness (AGI/ARI/ILI) occurrence.

### Acceptability and completeness

To evaluate acceptability, we calculated the proportion of invitees who answered the intake questionnaire. For completeness we calculated the proportion of responders and active responders for all weekly and follow-up questionnaires and examined the attrition. We used a *χ*^2^ test to compare the response proportion among participants.

### Representativeness

To assess representativeness, we used a *χ*^2^ test to compare cohort participants with the Swedish population in terms of sociodemographic characteristics (age, sex, county, education).

### Correlation with other syndromic surveillance systems

We calculated the AGI, ARI and ILI weekly incidence from week 48 (2013), to week 47 (2014), using the proportion of active participants fulfilling the case definition over all active participants. Age-specific incidence for AGI has been reported previously [8]. We directly standardized the incidence for the geographical location (North, Centre and South of Sweden) and for the age of the Swedish population, as it was reported on 31 December 2013 for the following age groups: <2, 2–4, 5–14, 15–39, 40–64, and ⩾65 years [9]. We considered reports fulfilling the case definitions for two consecutive weeks or more as one single episode. We excluded non-active responders and reports within 2 weeks from registration, as invitees may have been more motivated to join and report when ill.

We calculated the Spearman correlation coefficients (*r*) between: (*a*) AGI incidence and (i) the weekly proportion of samples positive for norovirus among tested samples, and (ii) the weekly proportion of queries on ‘winter vomiting disease’ to the 1177.se website, (*b*) ARI incidence and the weekly proportion of queries on ‘cough’ to the 1177.se website, (*c*) ILI incidence and (i) the weekly proportion of ‘fever in children’ as main reason for calling the 1177 hotline, and (ii) web query-based estimated (Get Well) incidence of people with ILI consulting a GP sentinel network [10, 11]. Before the analysis, we smoothed both Hӓlsorapport weekly incidences and data from other systems using a 2-week moving average.

We chose data, queries and reasons for calling that were already routinely used in surveillance programmes apart from queries on cough. We selected the query on cough based on the ARI case definition and no other query was tested. We performed the analysis, using Stata v. 13 software (Stata Corporation, USA)

### Participants’ experience

On 27 November 2014 (week 48, 2014), we sent out a final questionnaire to 3215 participants, to capture their experience of Hӓlsorapport. The questionnaire contained questions on reporting adherence, participation experience, willingness to extend participation, use of the provided email address to the Hälsorapport helpdesk, and satisfaction with the helpdesk and project website.

## RESULTS

### Acceptability

Of the 34 748 invitees, 3245 (9·3%) answered the intake questionnaire and were enrolled as participants.

Acceptability in terms of joining the cohort, differed across age groups, sex, county, and educational attainment (*P* < 0·001) (Table 1). Regarding age, the highest recruitment proportion was observed in those aged >64 years [10·4%, 95% confidence interval (CI) 9·4–11·4] and <5 years (10%, 95% CI 9·7–10·7) while invitees aged 15–39 years had the lowest recruitment proportion (6·3%, 95% CI 5·7–6·8). Compared to males, females also had a higher recruitment proportion (8·6%, 95% CI 8·2–9·0 *vs*. 10%, 95% CI 9·6–10·5). Acceptability increased with higher educational attainment: 3·3% (95% CI 2·8–3·8) in invitees with primary school education *vs*. 22% (95% CI 20·8–22·6) in invitees with at least 3 years of post-secondary education. Recruitment proportion across Swedish regions ranged between 8·7% and 9·6%.

**Table 1.** Sociodemographic characteristics of participants and people selected for invitation, Hälsorapport (n = 3245), Sweden 2013–2014

Sociodemographic conditions	Participants		Selected for invitation		Acceptance proportion		Total population		
	*N*	%	*N*	%	%	P*	*N*	%	*P*†
Age, years									
<5	1477	45	14 424	41	10	<0·001	579 019	6	<0·001
5–14	389	12	4355	12	9·3		1 067 082	11	
15–39	440	14	6768	19	6·3		3 055 723	32	
40–64	559	17	5686	16	9·8		3 070 833	32	
65–85	376	12	3609	13	10		1 872 207	19	
Total	3241	100	34 842	100	9·3		9 644 864	100	
Missing	4								
Sex									
Male	1524	47	17 766	51	8·6	0·005	4 814 357	50	<0·001
Female	1721	53	17 076	49	10		4 830 507	50	
Total	3245	100	34 842	100	9·3		9 644 864		
Area‡									
North	346	11	3953	11	8·7	0·252	1 157 135	12	0·02
Centre	1364	42	14 252	41	9·6		3 881 705	40	
South	1515	47	16 637	48	8·8		4 606 024	48	
Total	3225	100	34 842	100	9·3		9 644 864	100	
Missing	20								
Education§									
Primary school	188	5·8	5690	17	3·3	<0·001	1 355 236	19	<0·001
Upper secondary school	658	3·1	14 926	45	4·4		3 140 580	45	
Post-secondary school <3 years	613	19	4590	14	13		987 409	14	
Post-secondary school ⩾3 years	1765	55	8132	24	22		1 449 731	21	
Total	3224	100	33 338	100	9·7		6 932 956	100	
Missing	21		1504						

* Participants *vs.* invitees.

† Participants *vs.* total population.

‡ Education is for both participants and invitees; we recorded the education of the guardian for <16-year-olds.

§ North (Gävleborg, Norrbotten, Västerbotten, Jämtland, Västernorrland); Central (Stockholm, Uppsala, Södermanland, Dalarna, Västmanland, Örebro, Värmland); South (Östergötland, Jönköping,Västra Götaland, Halland, Skåne, Blekinge, Gotland, Kalmar, Kronoberg).

### Completeness

Of all participants, 98% joined the cohort before week 52 (2013), with the last participant joining in week 15 (2014). Over the study period, 30 (0·9%, 95% CI 0·6–1·3) participants asked to be removed from the cohort. The attrition was evenly distributed over the study period.

*Weekly questionnaires.* Responders answered 81% (*n* = 139 013) and active responders 79% (*n* = 134 419) of the weekly questionnaires sent between week 46 (2013) and week 47 (2014). The weekly median response proportion in active responders was 79% [interquartile range (IQR) 77–82%].

*Follow-up questionnaires.* Responders answered 90% (*n* = 16351, 95% CI 90·0–90·8) and active responders 88% (*n* = 15919, 95% CI 87·6–88·5) of the 18 085 follow-up questionnaires sent between week 50 (2013) and week 50 (2014). The weekly median response proportion in active responders was 88% (IQR 87–91%)

### Representativeness

Participants differed from the general population in terms of age, sex, geographical distribution, and educational attainment (*P* < 0·001, Table 2). When they joined the system, 58% of participants (1883, 95% CI 56·3–59·7) were aged <15 years, 53% (1721, 95% CI 51·3–54·8) were female and 56% (1811, 95% CI 54·1–57·6) lived in one of the three most densely populated counties, compared to 17%, 50% and 52% in the population, respectively. Of all participants, 74% (95% CI 72–76) had post-secondary education (the highest educational attainment of the first guardian was considered in case of participants aged <16 years, compared to 35% in the general population.

**Table 2.** End of follow-up questionnaire

Question (summarized)	Answers	*N*	%
Simplicity			
Complexity, weekly questionnaires	Simple	2428	99
	Difficult	28	1·1
	Other	7	0·3
Complexity, severity questionnaires	Simple	1558	84
	Difficult	238	13
	Other	62	3,3
Time to answer weekly questionnaires, if not sick	Little/right time	2427	99·2
	Too much time	9	0·4
	Other	12	0·5
Time to answer weekly questionnaires, if sick	Little/right time	2230	91
	Too much time	32	1·3
	Other	188	7·6
Time to answer severity questionnaires	Little/right time	1745	93·9
	Too much time	23	1·2
	I do not remember	91	4·9
Use of registered email address			
Type of email address used for Hälsorapport	Private	2080	88
	Professional	131	5·6
	Other	164	6·9
Use of email address when ill	Every day	1316	56
	A couple of times per week	580	25
	Once a week	221	9·3
	Less than once per week/never	129	5·4
	I am never sick	119	5·0
Adherence			
To have missed reporting ill episodes	Have reported all ill episodes	2303	94
	Have not reported all ill episodes	91	3·7
	I do not remember	56	2·3
To have interrupted reporting	Have interrupted reporting	108	4·4
	Have not interrupted reporting	1980	84
	Other	356	14·1
Reason for reporting interruption	Forgot	43	40
	Lack of motivation	17	16
	Busy	16	15
	Technical problems	14	13
	Not sick	9	8·3
	Other reasons	39	35·9
Considering to extend participation	Yes	1873	78
	No/I do not know	540	22·2

### Correlation with other syndromic surveillance systems

#### AGI

**Fig. 2.**
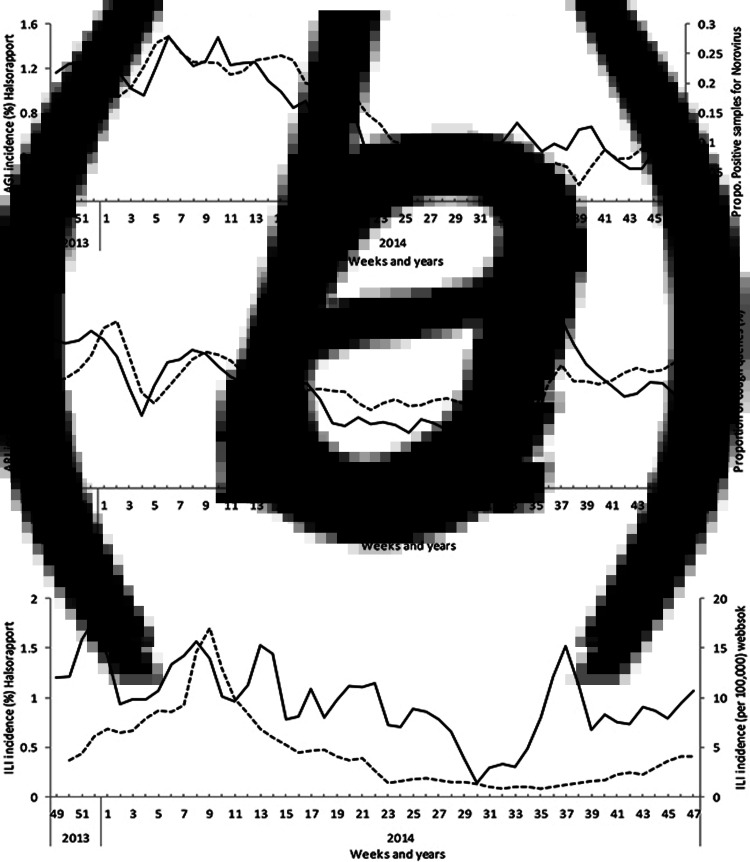
Continuous black line represents the age-standardized estimated (*a*) AGI, (*b*) ARI and (*c*) ILI incidence based on weekly questionnaires sent to Hälsorapport, a cohort of 3245 Swedish people, between November 2013 and November 2014. The dashed line represents the weekly proportion of samples positive for norovirus over the total sample tested for norovirus (voluntary and aggregated laboratory data submitted to the Public Health Agency of Sweden). (*b*) The weekly proportion of searches on cough over total searches launched to the Swedish medical website 1177.se. (*c*) The estimated weekly incidence of patients with ILI consulting sentinel GPs based on an algorithm applied to different search terms to the Swedish medical website 1177.se.

During the 52 weeks, 1208 (37%) participants reported at least one AGI episode. The Hӓlsorapport weekly AGI incidence correlated with weekly laboratory norovirus notification (*r* = 0·66, 95% CI 0·48–0·79) (Fig. 2*a*) and with the proportion of queries on winter-vomiting disease (*r* = 0·81, 95% CI 0·69–0·89).

#### ARI

During the 52 weeks, 2597 (80%) participants reported at least one ARI episode. The Hӓlsorapport weekly ARI incidence correlated with the proportion of queries on cough (*r* = 0·77, 95% CI 0·62–0·86) (Fig. 2*b*).

#### ILI

During the 52 weeks, 1142 (35%) participants reported at least one ILI episode. The Hӓlsorapport weekly ILI incidence correlated with ‘fever in children’ as the main reason for calling the 1177 hotline (*r* = 0·53, 95% CI 0·30–0·70) and ILI GP consultation estimate based on query data (Get Well) (*r* = 0·63, 95% CI 0·42–0·77) (Fig. 2*c*).

### Participants’ experience

Of the 3215 participants who received the final questionnaire, 77% and 72% (*n* = 2468 and 2329 participants) answered at least one question and completed the questionnaire, respectively. Of all respondents, 63% (*n* = 1537) reported their participation in the system as interesting and 89% (*n* = 2111) as meaningful.

In addition, 99% and 84% of responders described the weekly and follow-up questionnaires, respectively, as ‘easy to answer’ (Table 2). Responding to the weekly questionnaire in case of illness and to the follow-up questionnaire on severity took too much time for only 1·3% and 1·2% of responders, respectively.

In total, 4% of the participants reported having interrupted their reporting to the system or having missed reporting illness episodes. The mean number of episodes of illness that participants failed to report was 0·21 (s.d. = 2·17). Having forgotten to answer was the main reason for reporting interruption (40%).

Overall, 78% of responders replied that they would extend their participation in the system for a median time of 12 extra months (IQR 12–24 months). Of participants, 88% used private email accounts and more than 80% would check their email every day or more than once per week, even in case of illness.

## DISCUSSION

Overall, only a small proportion of invited people participated in Hӓlsorapport. However, its data completeness was high, with a limited number of individuals being lost to follow-up. Response proportions for the weekly and follow-up questionnaires were consistently high for the entire study period. Age-standardized AGI, ARI and ILI estimates correlated significantly with other surveillance data.

Hӓlsorapport required lengthy participation and completion of numerous questionnaires. This may have affected the acceptance to join the project and, consequently, the representativeness of the cohort. However, participation was similar to other recent cohort studies [12, 13], including the 2008–2009 UK Infectious Intestinal Disease study 2 (IID2) and the 2011–2012 Swedish study of work environment and disease epidemiology-infections (SWEDE-I), which had recruitment proportions ranging from 9% to 16%. However, it was lower than older, non-web-based European cohorts: the recruitment proportion of the Infectious intestinal disease study (IID1) conducted in the UK in the 1990s was 35% [14], and for the Sensor study [6] in The Netherlands in 1998–1999, it was 42%. In both latter studies, participants reported weekly the onset of gastrointestinal symptoms, but only for 6 months, which may have increased acceptance. In the IID1 study, family doctors signed the project invitations, while trained nurses followed-up the invitations with a telephone call or a reminder letter [14], which may also have increased participation. Overall, participation in epidemiological studies seems to have declined over the last decades [15, 16].

The invitation to participate in Hӓlsorapport was sent to an age-stratified random sample of the Swedish population. The main advantage of population-based cohort studies is the external validity, since selection biases are minimized if the recruitment proportion is high and the follow-up is stable. In Hӓlsorapport, the follow-up was consistently high, but the recruitment proportion was low even if in line with expectations. Even in population-based cohort studies, low recruitment proportion may limit the external validity [17], in particular if there is a considerable variation across sociodemographic conditions, as we observed in this cohort. However, unlike other syndromic surveillance systems, Hӓlsorapport enabled the assessment of its representativeness and since it is population-based, adjustments were possible. Furthermore, since the primary objective of Hӓlsorapport was to estimate the incidence of illnesses that are common in the community [18–20], the cohort stability was more important than its representativeness.

Participatory surveillance is a relatively recent approach that allows monitoring of specific conditions directly in the community by inviting volunteers from the general public to submit health-related information through web questionnaires [21]. This approach has been applied previously to address relevant public health questions [21]. However, since these studies are not population-based, they are not able to produce the community incidence of a specific syndrome. By contrast, because Hӓlsorapport is population-based, weighting of the results can produce incidence estimates.

Web-based panels have several limitations [21], with internet access being the most prominent one. In Sweden, internet usage has increased rapidly over the last decades and in 2013, 91% of the population used the internet at least once a week [22]. Furthermore, an independent report published in 2013 shows that the digital socioeconomic divides have largely disappeared in Sweden, remaining only in seniors and in relation to mobile technology [23]. These conditions minimize the bias due to non-coverage or lack of internet accessibility.

Hӓlsorapport participants reported checking their email accounts regularly, even though a small proportion reported doing it less than once per week when they were ill. This could have led to underreporting of illness. On the other hand, since participants who infrequently checked their email when ill represented a small minority, the related underreporting was likely negligible.

As in other similar studies, people with higher education were overrepresented, suggesting an underrepresentation of individuals with lower socioeconomic status. Even if low socioeconomic status is associated with higher risk of gastroenteritis in low-income countries [24], such an association has not been clearly demonstrated in high-income countries [25]. Studies aiming to examine potential associations between socioeconomic factors and respiratory infections, have mainly focused on severity and mortality, rather than disease occurrence [26]. As a result, it is difficult to speculate as to whether the socioeconomic misrepresentation may have led to an overestimation or an underestimation of these syndromes in the community, or if they have had an impact at all.

In Hӓlsorapport, children aged <5 years were overrepresented. This offered the opportunity to estimate the incidence of AGI, ARI and ILI more precisely in this age group, which is often difficult to recruit and one of the most affected in terms of severity and healthcare utilization [21, 27, 28].

Hӓlsorapport aimed to cover information gaps in the surveillance systems already in place; therefore there was no direct gold standard with which to validate its data. Nevertheless, age-standardized incidences correlated with other routinely used syndromic surveillance systems. These findings suggest that Hӓlsorapport offered a reasonable representation of temporal AGI, ARI and ILI patterns in the community. However, we found no clear peak in Hӓlsorapport ILI cases, although there was a clear peak in the ILI GP consultation estimate based on query data. This could potentially be because the GP consultation rate in Sweden may be affected by health-seeking behaviour of ILI patients when influenza is circulating and this knowledge may affect their likelihood of diagnosing patients as having ILI – which the web query-based consultation rate is designed to reflect. On the contrary, Hӓlsorapport is designed to generate a representation of ILI activity in the community, irrespective of healthcare-seeking behaviour and circulating infectious agents.

Participants found Hӓlsorapport to be easy and quick to use, which probably contributed to the low loss of follow-up and the high level of completeness. Furthermore, the intention of participants to continue their participation suggests that once recruitment has taken place, the cohort will continue reporting for more than 1 year.

As a result of this evaluation, Hӓlsorapport was restarted in 2015 with substantial differences compared with the previous edition. The invitation to participate was sent again to an age-stratified random sample of the population, but without applying any corrective factors for expected different acceptance proportions across age groups as it was done in 2013–2014. In order to increase acceptability and representativeness, participants were invited to answer only a monthly questionnaire on different public health issues. Based on resource prioritization, the description of which is outside the scope of this evaluation, routine monitoring of AGI, ARI, and ILI syndromes in the community was discontinued for the time being. Yet, given the high added value of such information, it was decided to retain the capacity to monitor weekly the occurrence of these conditions in case of health emergencies, such as pandemics.

### Limitations

It is difficult to draw far-reaching conclusions regarding the correlation findings, as the project had only been running for 1 year, and other surveillance systems do not measure the community incidence of the syndromes. In addition, we only standardized the weekly incidence for age and geographical distribution, although additional factors could have influenced our estimates. Moreover, we assessed the cohort representativeness only in terms of age, sex, area and education but we did not include several other factors that may influence the incidence of the syndromes under investigation (i.e. influenza vaccination, diet, lifestyle). Finally, other key attributes such as timeliness, stability and usefulness have not been the object of this evaluation, even if they could have provided useful elements to fully understand the strengths and weaknesses of the system.

## CONCLUSIONS

This evaluation provides insights into key attributes of a population- and web-based syndromic surveillance project that aimed to estimate the burden of AGI, ARI and ILI in the community. We found that acceptability of Hӓlsorapport was low and representativeness not optimal. Yet, completeness was high and participants showed high motivation and most participated actively throughout the year. The correlation with other syndromic surveillance systems suggests that Hӓlsorapport is a useful tool to complement traditional and syndromic surveillance systems and to estimate the burden of AGI, ARI and ILI in the community. However, since the system is web- and population-based, the generalizability of our results is likely limited to high-income countries with population registers and high internet access.

## Supplementary material

For supplementary material accompanying this paper visit http://doi.org/10.1017/S0950268816003290. click here to view supplementary material
