# Supplementary material for: High added value of a population-based participatory surveillance system for community acute gastrointestinal, respiratory and influenza-like illnesses in Sweden, 2013–2014 using the web
Source: Epidemiol Infect. 2017 Jan 31;145(6):1193–202. doi: 10.1017/S0950268816003290 (PMC5426337; doi:10.1017/S0950268816003290)
Supplement: Supplementary file 1 [file S0950268816003290sup001.docx]

Epidemiology and Infection

High added value of a population-based participatory surveillance system for community acute gastro-intestinal, respiratory and influenza-like illnesses in Sweden, 2013-2014 using the web.

A. Pini, H. Merk, AS. Carnahan, I. Galanis, E. van Straten, K. Danis, M. Edelstein, A. Wallensten

Supplementary material

S1. List of symptoms investigated through weekly questionnaires

- Fever
- Chills
- Sneezing
- Sore throat
- Runny nose
- Cough
- Shortness of breath/Difficulty breathing
- Feeling tired or exhausted
- Muscle/join pain
- Headache
- Stomach ache
- Abdominal cramps
- Nausea
- Vomiting
- Diarrhoea
- Mucus in stool
- Blood in stool
- Other
